# Club Rates

**Published:** 1884-07

**Authors:** 

**Affiliations:** Atlanta, Ga.


### READ THIS.—CLUB RATES.

In another place in this issue will be found an announcement of a reduction of the regular price of THE JOURNAL to $1.50, for thirty days. This discount is made to induce those to whom sample copies are sent to become permanent readers. THE JOURNAL hopes to merit the support of all who subscribe.

*The Southern Cultivator and Dixie Farmer*, a large and splendid agricultural and industrial journal, price $1.50 per annum, will be sent with THE JOURNAL for $1.00 during the next thirty days.

*The Christian Index*, a large 16-page family, religious journal—one of the oldest and best in the land—price $2.50, will be sent with THE JOURNAL for $1.50 during the next thirty days.

Or we will, for $1 00 additional, send *Common Sense in the Kitchen*, a most valuable book to housekeepers, containing about 400 pages of recipes. This book is neatly bound in cloth.

For $1 00 extra we will also send THE BOOK OPENED, or, the Analysis of the Bible, bound neatly and substantially in cloth, and containing nearly 400 pages. This is a most interesting review of the sacred scriptures and will be appreciated by all who love the Bible.

Or for 10 cents extra we will send either of the following, bound in paper; viz.: Robinson Crusoe, Arabian Nights, Pilgrim's Progress.

Sample copies of the JOURNAL, the *Southern Cultivator* and the *Christian Index* sent free on application.

We offer them at the low rates specified, because we are anxious that all should subscribe for and read them.

Remember this offer is for only 30 days. Address,

JAS P. HARRISON & Co., *Publishers.*

Atlanta, Ga.

